# Cytochrome P450 2D-mediated metabolism is not necessary for tafenoquine and primaquine to eradicate the erythrocytic stages of *Plasmodium berghei*

**DOI:** 10.1186/s12936-016-1632-8

**Published:** 2016-12-07

**Authors:** Erin E. Milner, Jonathan Berman, Diana Caridha, Samuel P. Dickson, Mark Hickman, Patricia J. Lee, Sean R. Marcsisin, Lisa T. Read, Norma Roncal, Brian A. Vesely, Lisa H. Xie, Jing Zhang, Ping Zhang, Qigui Li

**Affiliations:** 1 Walter Reed Army Institute of Research (WRAIR), Silver Spring, MD USA; 2 United States Army Medical Materiel Development Authority (USAMMDA), Ft Detrick, Frederick, MD USA

## Abstract

**Background:**

Due to the ability of the 8-aminoquinolines (8AQs) to kill different stages of the malaria parasite, primaquine (PQ) and tafenoquine (TQ) are vital for causal prophylaxis and the eradication of erythrocytic *Plasmodium* sp. parasites. Recognizing the potential role of cytochrome (CYP) 450 2D6 in the metabolism and subsequent hepatic efficacy of 8-aminoquinolines, studies were designed to explore whether CYP2D-mediated metabolism was related to the ability of single-dose PQ and TQ to eliminate the asexual and sexual erythrocytic stages of *Plasmodium berghei.*

**Methods:**

An IV *P. berghei* sporozoite murine challenge model was utilized to directly compare causal prophylactic and erythrocytic activity (asexual and sexual parasite stages) dose–response relationships in C57BL/6 wild-type (WT) mice and subsequently compare the erythrocytic activity of PQ and TQ in WT and CYP2D knock-out (KO) mice.

**Results:**

Single-dose administration of either 25 mg/kg TQ or 40 mg/kg PQ eradicated the erythrocytic stages (asexual and sexual) of *P. berghei* in C57BL WT and CYP2D KO mice. In WT animals, the apparent elimination of hepatic infections occurs at lower doses of PQ than are required to eliminate erythrocytic infections. In contrast, the minimally effective dose of TQ needed to achieve causal prophylaxis and to eradicate erythrocytic parasites was analogous.

**Conclusion:**

The genetic deletion of the CYP2D cluster does not affect the ability of PQ or TQ to eradicate the blood stages (asexual and sexual) of *P. berghei* after single-dose administration.

**Electronic supplementary material:**

The online version of this article (doi:10.1186/s12936-016-1632-8) contains supplementary material, which is available to authorized users.

## Background

Primaquine (PQ) continues to be an indispensable drug for radical cure and presumptive anti-relapse therapy (PART or terminal prophylaxis) of *Plasmodium vivax* and *Plasmodium ovale* infections, while serving as an alternative for primary prevention [1, 2]. PQ is active against the hepatic stages of all human malaria parasites and the only FDA-approved drug active against the dormant hypnozoite stages of *P. vivax* and *P. ovale* [3]. While there is no clinical benefit to the patient, PQ can be used as a control measure to render *Plasmodium falciparum* sexual blood stages *(*gametocytes) non-infective to mosquitoes and, therefore, disrupt transmission [4].

Tafenoquine (TQ) is a long half-life PQ analog that eliminates both hepatic and erythrocytic stages of *Plasmodium* sp. and is currently being developed by the U.S. Army for chemoprophylaxis of all malaria and by GlaxoSmithKline and Medicines for Malaria Venture for the radical cure of *P. vivax.* [5, 6] PQ and TQ are generally well tolerated, but there are known liabilities associated with hemolytic reactions in patients with glucose-6-phosphate dehydrogenase (G6PD) deficiencies [2, 6–10]. Patients should be tested for G6PD deficiency before the multi-dose regimens of PQ are prescribed for radical cure, terminal prophylaxis, or chemoprophylaxis. The WHO recommends a single administration of 0.25 mg base/kg PQ as a relatively well-tolerated (even in G6PD deficient individuals) gametocytocidal dose to inhibit the transmission of *P. falciparum* malaria [4].

In addition to this potential liability leading to the need for G6PD testing prior to dosing, the concept of PQ and TQ as prodrugs in the context of causal prophylaxis and radical cure has introduced another layer of complexity with administration. Recent pre-clinical findings at the Walter Reed Army Institute of Research (WRAIR) have shown that PQ requires CYP2D-mediated metabolism for *Plasmodium berghei* causal prophylaxis in mice [11, 12]. Tafenoquine was also shown to require CYP2D-mediated metabolism for causal prophylactic activity in *P. berghei*-infected mice [13]. These findings have led some to suggest an 8-aminoquinoline class effect associated with CYP2D-mediated metabolism requirements for activity [14]. Given that nearly ten percent of certain populations are cytochrome P450 (CYP) 2D6 deficient [15], it is clinically important to investigate whether CYP2D-mediated metabolism is related to the ability of PQ and TQ to eradicate the asexual and sexual erythrocytic stages of *Plasmodium* sp.

To probe this relationship, an established murine *P. berghei* challenge model was utilized involving an intravenous (IV) *P. berghei* sporozoite inoculation on day 0 that results in a hepatic infection followed by subsequent erythrocytic infection on day 3 [16, 17]. Vehicle control mice are generally deceased or euthanized due to morbidity within a week. The utility of this model has also been demonstrated for non-8-aminoquinoline structural motifs [16]. As shown in Table 1, administering drugs days −1, 0, and/or +1 versus day +4 post infection allows for the direct comparison of hepatic causal prophylactic activity versus anti-erythrocytic activity within the same challenge model. The utilization of luciferase-expressing sporozoites from *P. berghei*-infected mosquitoes and an in vivo imaging system (IVIS) permits visualization and quantitation of developing hepatic stages of *P. berghei* for the causal prophylactic assessment of drugs, while flow cytometry (parasitaemia) and microscopy (gametocytaemia) are used to assess erythrocytic infections. To assess the effect of CYP2D-mediated metabolism on the ability of PQ or TQ to eradicate erythrocytic infections, C57BL wild-type (WT) mice were utilized along with knock-out (KO) mice purchased with the murine CYP2D gene cluster deleted and monitored parasitaemia and gametocytaemia [18].

**Table 1:** In vivo *P. berghei* intravenous (IV) sporozoite challenge causal prophylactic and erythrocytic models

Model	Causal prophylactic model	Erythrocytic treatment model
Drug administration (day)^a^	−1, 0, and/or +1	+4
Endpoint^b^	Hepatic and erythrocytic parasites (asexual parasitemia)	Erythrocytic parasites: parasitemia (asexual), gametocytemia (sexual)
Technique	IVIS (liver), flow cytometry (parasitemia)	Flow cytometry (parasitemia) microscopy (gametocytemia)

^a^Day of drug administration relative to IV sporozoite inoculation (day 0). *IVIS* In vivo imaging system

^b^Animals are monitored for 30 days or recrudescence and subsequent euthanasia due to comorbidities

## Methods

### Study drugs

Primaquine (WR002975) and tafenoquine (WR238605) were supplied by the Walter Reed Army Institute of Research (WRAIR) chemical repository. Primaquine (PQ) was supplied as the bisphosphate salt and tafenoquine (TQ) was supplied as the succinate salt. Dosing was calculated based on the milligram per kilogram (mg/kg) body weight with respect to the molecular weight of the free base. The bulk drug of TQ used for the study was synthesized for the WRAIR by Ash Stevens, Inc. (Detroit, MI USA) with a purity of >98%. PQ was purchased from Sigma Aldrich (St. Louis, MO USA) with a purity of >98%.

### Sporozoites and viability

Luciferase-expressing *P. berghei* (strain ANKA) sporozoites [19] were obtained from laboratory-reared female *Anopheles stephensi* mosquitoes from the Department of Entomology, Walter Reed Army Institute of Research. The mosquitoes were maintained at 18°C for 17–22 days after feeding on *P. berghei* malaria infected Swiss ICR mice. As the salivary glands were extracted from the malaria-infected mosquitoes, they were stored on ice in RPMI medium with 1% mouse serum. Sporozoites were recovered by the method of Ozaki [20]. To ensure the inoculated sporozoites were viable following the isolation procedure, they were stained with a vital dye containing fluorescein diacetate [50 mg/mL acetone; Sigma Aldrich (St. Louis, MO USA)] and ethidium bromide [20ug/mL in phosphate-buffered saline; Sigma Aldrich (St. Louis, MO)] and counted in a haemocytometer. The viability of sporozoites ranged from 87 to 100%.

### Animal housing

Male 8–12 week-old C57BL CYP2D KO mice, bred using the method Scheer and colleagues [18], were purchased from Taconic (Hudson, NY USA). Male 6–8 week-old C57BL/6 wild-type mice were purchased from Charles River Laboratories (shipped from Raleigh, NC USA). Upon arrival, all animals were acclimated for 7 days in quarantine. The animals were housed in a cage contained in a room with a temperature range of 64–79 °F, 34–68% relative humidity, and a 12 h light/dark cycle. Food and water were provided ad libitum during quarantine and throughout the study. The animals were fed a standard rodent maintenance diet. The C57BL/6 WT and C57BL CYP2D KO mice were cautiously housed separately and identified with the appropriate cage cards. Extra care was taken to ensure there were no labelling errors associated with WT and KO mice.

### Intravenous sporozoite inoculation

Sporozoites were rapidly isolated from the same batch of mosquitoes for a given experiment and administered to C57BL/6 wild-type (WT) and CYP2D knock-out (KO) mice the same morning to control for biological and time-dependent variability in sporozoite preparations. Each mouse was inoculated intravenously in the tail vein with 10,000 sporozoites suspended in 0.1 mL volume on day 0. The WT group was randomized separately from the KO group after sporozoite inoculation and prior to dosing with the test drugs or vehicle control. All animal studies were performed under IACUC-approved protocols. All animal experiments were conducted in a facility accredited by the Association for the Assessment and Accreditation of Laboratory Animal Care, International, and in compliance with the Animal Welfare Act and other federal statutes and regulations relating to animals and experiments involving animals and adhere to the principles stated in the Guide for the Care and Use of Laboratory Animals (National Academy Press, 1996).

### Genetic verification

Ear punch pathological samples were collected from a subset of WT and KO mice and stored in 70% ethanol at −80 °C for 12 h in preparation for shipment the following day. The samples were blinded with identification numbers and shipped with dry-ice overnight to Taconic, where the biopsies were transferred to a 96-well box and the DNA was extracted using the Qiagen Blood and Tissue extraction Kit^®^ (Hilden, Germany). After extraction, the DNA was evaluated using a spectrophotometer to determine the concentration and normalized to 10 ng/µL. A master mix was prepared using reagents obtained through Qiagen, dNTPs, and the four primers from MWG Operon. The master mix was distributed into the PCR plate, and upon thermal cycling, the product was processed with a capillary electrophoresis instrument. Reagent, primer information, and assay data are available in the Additional file 1: Figures S1–S4.

### Test agent administration

Drug substances were administered orally on days −1, 0, and/or +1 (causal prophylactic experiments) or on day 4 (blood-stage experiments) with respect to sporozoite inoculation on day 0. Dosing calculations were based upon body weight measurements obtained on the day of dosing prior to preparing fresh stock solutions of the test compounds by grinding the needed quantity of drug in cold (4 °C) 0.5% (w/v) hydroxyethyl cellulose and 0.2% (v/v) Tween-80 (0.5% HECT) and diluting to the appropriate concentration. Drugs were ground using a ProScientific 300D homogenizer (Oxford, CT) and the particle size was measured using a Horiba LA-950V2 particle size analyzer (West Chicago, IL). Oral doses were delivered via an intragastric feeder (18-gauge) to the designated recipient.

### In vivo imaging system (IVIS)

In vivo imaging studies of bioluminescence activity from luciferase expressing *P. berghei* infected mice were performed using a Perkin Elmer IVIS Spectrum (Hanover, MD). Mice were evaluated at 24 h post sporozoite inoculation to confirm a liver-stage malaria infection. Mice received 200 mg/kg luciferin (Gold Biotechnology, St. Louis, MO) intraperitoneally in a volume not to exceed 200 µL. Five minutes post luciferin administration, the mice were anesthetized in a sleeping chamber with inhaled isoflurane. The mice were then positioned ventral side-up on the IVIS on a 37 °C platform. The mice continue to receive isoflurane through nose cone delivery. A camera exposure time of 5 min was utilized for the 24, 48, and 72 h time points with f-stop = 1 and large binning setting. Quantitative analysis of bioluminescence emitted from whole bodies or region of intensity (ROI) were determined by measuring the luminescence signal intensity in photons/second using the ROI settings of the Living Image^®^ 4.0 software. The ROI, which measurements were expressed in total flux of photons, was set to measure the bioluminescence signal emitted in the abdominal area at the location of the liver (24 and 48 h) and/or whole body imaging at 72 h.

### Flow cytometry (FCM)

Mice were analysed for asexual blood stage infections by determination of parasitaemia of tail blood samples (3 μL each) using a FC500 MPL flow cytometer (Beckman-Coulter Co., CA USA), which conducts five-color analysis from either single or dual laser excitation. Infected erythrocytes, uninfected erythrocytes, and leukocytes were gated on logarithmic forward/side dot plots. Cells were analysed at an average rate of 2000–3000 erythrocytes/s. Filters were placed before the green (FL-1) and red (FL-2) photomultiplier tubes (PMTs) such that the green PMT registered fluorescence emission between 520 and 555 nm, and the red PMT measured emission greater than 580 nm. 2–3 µL of blood from the mouse tail was collected directly into 0.3 mL of 1% heparinized isotonic buffer (PBS saline). In this study, 1 mL 0.04% of glutaraldehyde was used for fixation and the samples were then incubated at 4 °C for 60 min. The cells were centrifuged at 450×*g* for 5 min. The supernatant was removed by aspiration and the cells were re-suspended in 0.5 mL PBS buffer supplemented with 0.25% (v/v) Triton X-100 for 10 min incubation at room temperature. After centrifugation, the permeabilized cells were re-suspended in 0.5 mL of RNase at 1 mg/mL concentrations and incubated for at least 2 h at 37 °C to ensure complete digestion of reticulocytes. *Plasmodium berghei* infection in mice results in anaemia and subsequent reticulocytosis. Therefore, high RNAse concentrations for digesting large amounts of reticulocytes RNA were required for assessment of parasitaemia in this mouse model. YOYO-1 dye (from 1 mM stock solution in DMSO) was diluted to 2500 ng/mL (100-fold) concentrations in PBS and 20 µL of YOYO-1 solution at 2500 ng/mL was added to 0.5 mL of sample to a final dye concentration of 100 ng/mL of YOYO-1, which has been shown to be optimal to discriminate infected erythrocytes from the lowest (0.01%) to the highest parasitaemia counts (74.0%) [21, 22]. It should be noted that tail blood flow cytometry measurements were performed on 10 naïve mice which all resulted in a background flow cytometry value of 0.3% parasitaemia. This background value was subtracted from the flow cytometry values obtained for each animal during the study. All values in this manuscript reflect corrected flow parasitaemia with 0.3% background subtracted.

### Slide preparation and light microscopy

With the mouse restrained, a needle was used to prick the tail vein and one drop of blood was massaged from the vein and directly placed on a 25 × 75 mm microscopy slide (Fischer Scientific, USA) labelled with the date and animal ID number. A clean slide was used to “smear” the blood to create a single layer of erythrocytes/reticulocytes with a “feathered-edge.” The thin smear was allowed to dry at ambient room temperature for 4 h prior to fixing the slides with methanol. 20% Giemsa (Sigma Aldrich, USA) in phosphate buffer [10L batch: 7 g potassium phosphate monobasic (Fischer Scientific, USA), 10 g sodium phosphate dibasic (Fischer Scientific, USA), 10L DI water] was utilized to stain the slides for 30 min prior to rinsing with DI water. The stained slides were allowed to dry 12 h in ambient temperature prior to adhering cover slips (Fischer Scientific, USA) using Poly-Mount^®^ (Polysciences Inc., Warrington, PA). The slides were randomly distributed to 4 microscopists who were provided guidelines for slide reading methodology. Using a 100× high-power microscope, an eyepiece graticule with a St Andrew’s cross, and a layer of immersion oil (Sigma Aldrich, USA), parasitized cells in ten fields were quantified to provide the mean cell percentages associated with the sexual and asexual stages of *P. berghei* [23–25]. Percent parasitaemia was calculated as [(mean number asexual parasites)/(mean number of total RBCs in one quadrant × 4)] × 100. The percent gametocytaemia was calculated as [(mean number mature gametocytes)/(mean number of total RBCs in one quadrant × 4)] × 100.

### Parasitemia correlation

The quantification of erythrocytic parasites was accomplished using flow cytometry (asexual stages) and microscopy (sexual and asexual parasites). The utilization of flow cytometry allowed for the rapid determination of parasitaemia (asexual parasites) and the results are reflected in the tables and figures of the manuscript. Due to the meticulous nature of the microscopists, both sexual and asexual parasites were quantified. The percent parasitaemia derived from microscopy and flow cytometry was subsequently utilized to correlate the two methods. The relationship between percent parasitaemia determined using flow cytometry versus light microscopy was performed using R version 3.2.4. Linear and non-linear relationships were analysed utilizing linear and polynomial regression as well as a repeated measures analysis with a spatial power law covariance structure to account for correlation between time points. The correlation serves to link the parasite quantification derived from flow cytometry and microscopy. Correlation results are presented in the Additional file 1: Figure S7.

### Pharmacokinetic parameter determination

Pharmacokinetic (PK) parameters for PQ and TQ in plasma and liver generated by the WRAIR [26, 27] were analysed using noncompartmental analysis (NCA) via the Phoenix-Win-Nonlin software package (version 6.4; Pharsight Corp., Mountain View, CA). The PK parameters were utilized to explore the variability between wild-type and CYP2D knock-out mice.

### Statistical analysis

All tests of significance between groups across days were performed based on a linear regression model with repeated measures fit using a spatial power law covariance matrix (similar to the AR(1) model but allowing for unequally spaced days). All models included main effects for treatment group and study day, as well as a group × day interaction. When comparing mouse strains, the model also included a main effect for strain, the two-way interactions with group and day, and the three-way group × day × strain interaction. Significance was based on the means from the interaction terms within day, and correction for multiple testing was based on Tukey’s post hoc correction for all pairwise tests. Correlation was analysed based on a simple linear regression model, polynomial regression models up to third-degree polynomials, and a repeated measures model. A LOESS curve was also fit for comparison.

### Graphical representations

Data analysis and graphical representations were generated using either Phoenix-Win-Nonlin software package (version 6.4; Pharsight Corp., Mountain View, CA), Microsoft Excel, and R version 3.2.4.

## Results

### C57BL/6 wild-type (WT) murine causal prophylactic and treatment dose-ranging studies

Single-dose primaquine (PQ) and tafenoquine (TQ) dose-ranging studies were completed in order to assess the dose–response relationships between causal prophylactic (hepatic) activity and treatment (erythrocytic) activity and establish the dosing regimen for the CYP2D KO versus WT study erythrocytic study.

#### Primaquine causal prophylactic (hepatic) activity

PQ was administered for causal prophylaxis on either day 0 and/or day +1 relative to IV sporozoite inoculation on day 0. Traditionally, the WRAIR administers drugs on days −1, 0, +1 to assess causal prophylactic activity in the IV sporozoite challenge model [16, 17]. Given the short half-life and rapid clearance of PQ in C57BL wild-type mice (plasma t_1/2_ = 1.5 ± 0.4 h; liver t_1/2_ = 3.6 ± 1.2 h [26]), it was predicted that administering PQ on day −1 would not result in the clearance of hepatic parasites on day 0 and was not necessary for causal prophylaxis. This was confirmed when 25 mg/kg of PQ was administered day 0 and day +1 leading to 100% suppression of the hepatic IVIS signal and no detected erythrocytic infection after 30 days post infection. Subsequently single-dose PQ (40, 20, or 10 mg/kg) was administered day 0 relative to IV sporozoite inoculation. The 40 and 20 mg/kg cohorts led to 100% suppression of the hepatic IVIS signal and no detected erythrocytic infection. The 10 mg/kg cohort led to 100% suppression of the hepatic IVIS signal and no erythrocytic infection was detected in 3/5 mice. This suggests that hepatic parasites below the IVIS limit of detection exited the liver to infect the blood. The administration of 40 mg/kg of PQ day +1 led to incomplete suppression of the hepatic IVIS signal and 0/5 animals free of parasitaemia (Table 2).

**Table 2:** Primaquine (PQ) wild-type (WT) dose-ranging studies using C57BL/6 mice

Day dose administered^a^	PQ dose (mg/kg)	No hepatic IVIS signal^b^	No asexual erythrocytic infection (parasitemia)^c^	No sexual erythrocytic infection (gametocytemia)^c^
Causal prophylactic model				
D0, D1	25 × 2d	5/5 (D1), 5/5 (D2)	5/5	NA
D0	10	5/5 (D1), 5/5 (D2)	3/5	NA
	20	5/5 (D1), 5/5 (D2)	5/5	NA
	40	5/5 (D1), 5/5 (D2)	5/5	NA
D1	40	1/5 (D1), 1/5 (D2)	0/5	NA
Erythrocytic treatment model				
D4	10	NA	0/5	0/5
	20	NA	0/5	0/5
	40	NA	0/5	0/5

*PQ* primaquine, mg/kg milligrams free base of drug per kg body weight, *NA* not applicable, *IVIS* in vivo imaging system

^a^Day of drug administration relative to intravenous sporozoite (IV SPZ) challenge (day 0) using C57BL/6 wild-type mice

^b^Number of animals without an IVIS signal (indicates no hepatic infection). *D1* day 1 (relative to IV SPZ challenge day 0). *D2* day 2 (relative to IV SPZ challenge day 0)

^c^Number of animals without parasitemia (flow cytometry) or gametocytemia (microscopy) day 29. N = 5 animals per cohort

#### Primaquine erythrocytic treatment activity

Single-dose PQ (10, 20, and 40 mg/kg) was administered on day +4 relative to IV sporozoite inoculation and blood samples were analysed using flow cytometry and light microscopy to assess parasitaemia and gametocytaemia, respectively. PQ administration in each cohort led to the initial eradication of erythrocytic parasites followed by recrudescence observed on day 11. These suggest PQ eradicates hepatic infections at lower doses than are required to eliminate erythrocytic infections (Table 2).

#### Tafenoquine causal prophylactic (hepatic) activity and erythrocytic treatment activity

TQ exhibited comparable dose–response relationships for hepatic and erythrocytic reservoirs of parasitaemia in wild-type animals. As shown in Table 3, 20 mg/kg TQ led to 100% suppression of the IVIS signal and no detectable erythrocytic infection for 5/5 mice when administered on days −1, 0, and/or +1, while lower doses led to either incomplete suppression of the IVIS signal or failure to eradicate the erythrocytic infections within the dosing cohorts. Given the long half-life of TQ in wild-type mice (plasma t_1/2_ = 53.8 ± 3.5 h; liver t_1/2_ = 83.5 ± 2.3 h [27]), the causal activity of TQ administered on day −1 is likely related to drug remaining in the liver when sporozoites are inoculated on day 0; the anti-erythrocytic activity in the absence of causal activity of TQ administered on day +1 could be related to drug remaining in the circulation when parasites emerge from the liver on day +3. The administration of 20 mpk day +4 led to the eradication of erythrocytic parasites in 4/5 mice with recrudescence observed in 1/5 mice after 29 days (Table 3).

**Table 3:** Tafenoquine (TQ) wild-type (WT) dose-ranging studies using C57BL/6 mice

Day dose administered^a^	PQ dose (mg/kg)	No hepatic IVIS signal^b^	No asexual erythrocytic infection (parasitemia)^c^	No sexual erythrocytic infection (gametocytemia)^c^
Causal prophylactic model				
D1	2.5	0/5 (D1), 0/5 (D2)	0/5	NA
	5	3/5 (D1), 2/5 (D2)	0/5	NA
	10	5/5 (D1), 5/5 (D2)	4/5	NA
	20	5/5 (D1), 5/5 (D2)	5/5	NA
D0	2.5	0/5 (D1), 0/5 (D2)	0/5	NA
	5	4/5 (D1), 4/5 (D2)	3/5	NA
	10	3/5 (D1), 4/5 (D2)	2/5	NA
	20	5/5 (D1), 5/5 (D2)	5/5	NA
D1	20	0/5 (D1), 0/5 (D2)	5/5	NA
Erythrocytic treatment model				
D4	5	NA	0/5	0/5
	10	NA	0/5	0/5
	20	NA	4/5	4/5

*TQ* tafenoquine, *mg/kg* milligrams free base of drug per kg body weight, *NA* not applicable, *IVIS* in vivo imaging system

^a^Day of drug administration relative to intravenous sporozoite (IV SPZ) challenge (day 0) using C57BL/6 wild-type mice

^b^Number of animals without an IVIS signal (indicates no hepatic infection). D1 = day 1 (relative to IV SPZ challenge day 0). D2 = day 2 (relative to IV SPZ challenge day 0)

^c^Number of animals without parasitemia (flow cytometry) or gametocytemia (microscopy) day 29. N = 5 animals per cohort

### Comparison of PQ or TQ in wild-type (WT) and CYP2D knock-out (KO) C57BL mice

The disease progression of *P. berghei* in both liver and blood was shown to be analogous in C57BL wild-type (WT) and CYP2D knock-out (KO) mice (refer to Additional file 1: Figure S3). Following IV sporozoite inoculation (day 0), the presence of a liver stage infection was detected at 24 and 48 h followed by an erythrocytic infection at 72 h in both WT and CYP2D KO mice. The erythrocytic infection progressed analogously in WT and CYP2D KO vehicle control mice until mortality or euthanasia due to morbidity occurred within approximately one week following infection.

A single dose of drug was administered day +4 post infection to study the effect of CYP 2D-mediated metabolism relative to the ability of PQ and TQ to initially eradicate *P. berghei* erythrocytic stages (rather than determine the optimal dosing regimen to avoid recrudescence). Blood samples were analysed using flow cytometry (asexual stages) and light microscopy (sexual stages) to assess parasitaemia (Additional file 1: Figure S5) and gametocytaemia (mature gametocytes; Additional file 1: Figure S6), respectfully.

**Fig. 1:**
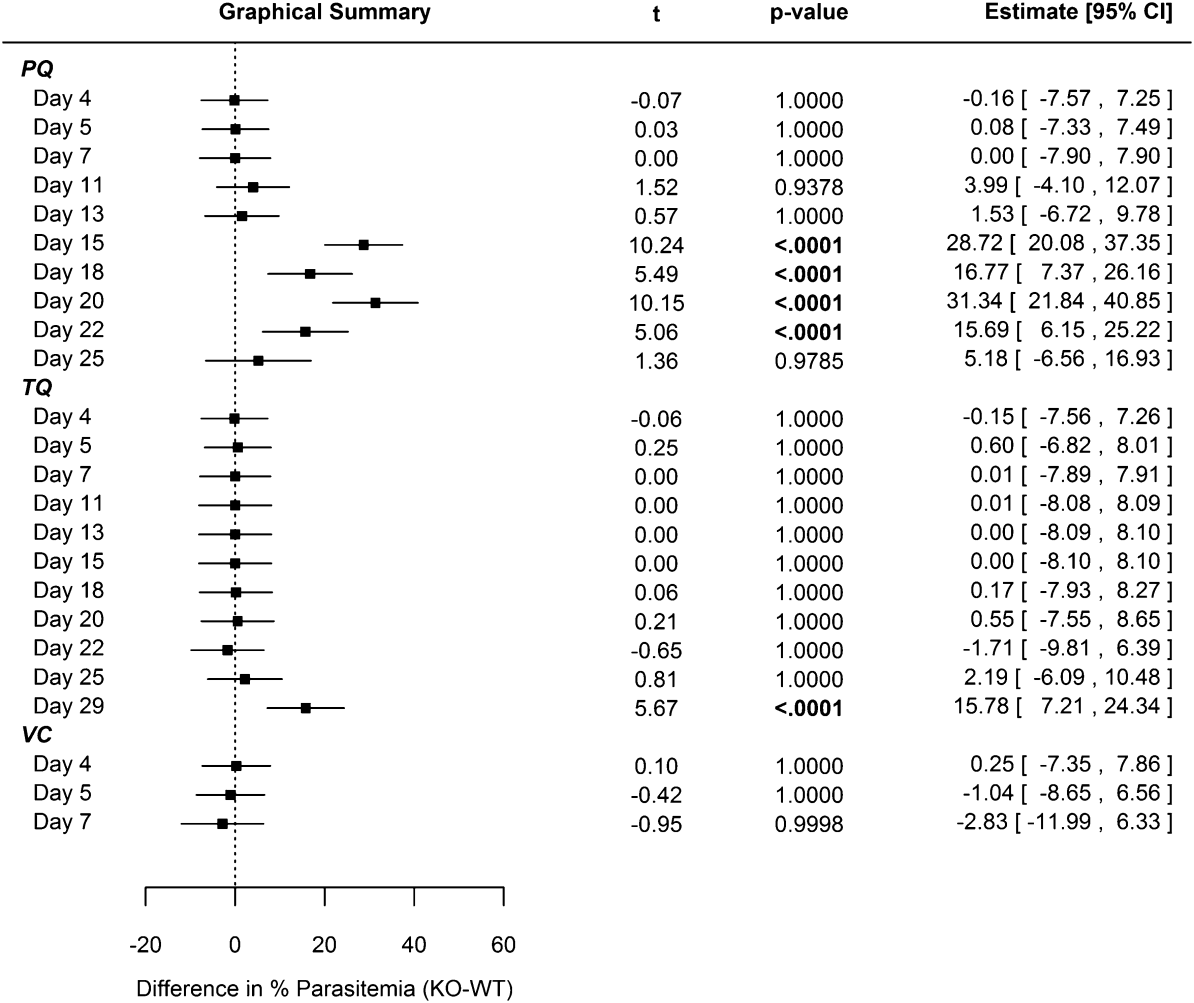
Statistical comparison regarding differences in parasitemia between WT and CYP2D KO mice. The data were modeled using linear regression with repeated measures with a spatial power law covariance structure. The model included terms for study day, treatment group, mouse strain, and all interactions between the three terms. All point estimates, intervals, and p values therefore represent the differences within each day between mouse strains after adjusting for repeated measurements on the same mice. 40 mg/kg primaquine (*PQ*), 25 mg/kg tafenoquine (*TQ*), or vehicle control (*VC*) administered day 4 relative to intravenous sporozoite (*IV SPZ*) challenge (day 0) using C57BL/6 wild-type or C57BL/6 CYP2D knock-out mice. N = 10 animals per cohort. (Refer to Additional file 1: Figure S5 for percent parasitemia)

**Fig. 2:**
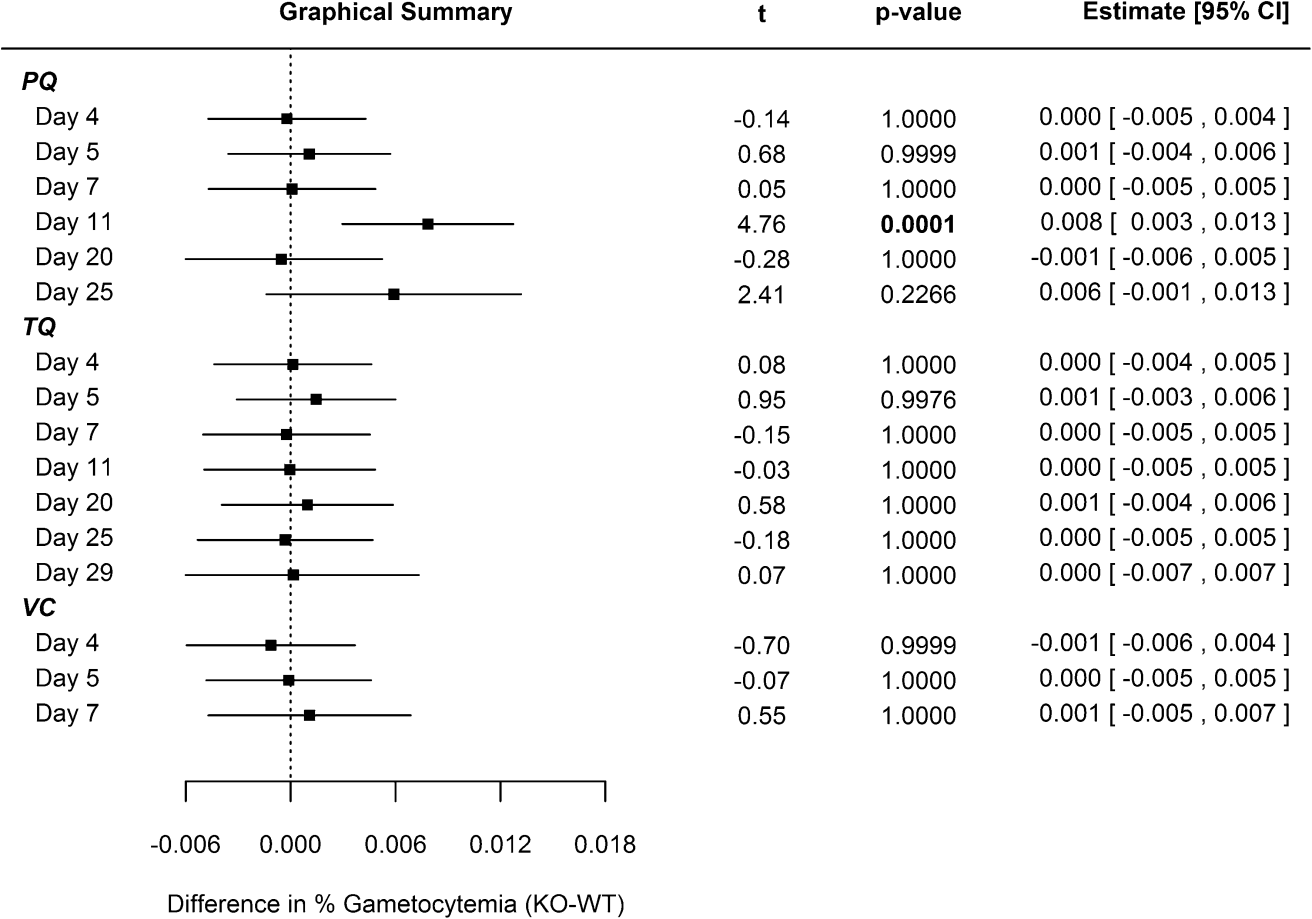
Statistical comparison regarding differences in gametocytemia between WT and CYP2D KO mice. The data were modeled using linear regression with repeated measures with a spatial power law covariance structure. The model included terms for study day, treatment group, mouse strain, and all interactions between the three terms. All point estimates, intervals, and p values therefore represent the differences within each day between mouse strains after adjusting for repeated measurements on the same mice. 40 mg/kg primaquine (*PQ*), 25 mg/kg tafenoquine (*TQ*), or vehicle control (*VC*) administered day 4 relative to intravenous sporozoite (*IV SPZ*) challenge (day 0) using C57BL/6 wild-type or C57BL/6 CYP2D knock-out mice. N = 10 animals per cohort. (Refer to Additional file 1: Figure S6 for gametocytemia)

Single-dose PQ (40 mg/kg) and TQ (25 mg/kg) eradicated erythrocytic parasites in both WT and CYP2D KO mice for one week post-drug administration. Recrudescence was observed day 11 in the PQ-treated WT and CYP2D KO cohorts (Additional file 1: Figures S5 and S6), which corresponds to the short half-life (WT t_1/2_ = 1.5 ± 0.4 h; CYP2D KO t_1/2_ 3.5 ± 0.9 h) and rapid clearance of PQ in plasma [26]. Comparing CYP 2D KO versus WT cohorts, no statistically significant differences in mean percent parasitaemia (Fig. 1) were observed for PQ until day 15, which resolved by day 25 just prior to euthanasia. In regards to gametocytaemia, differences between PQ-treated WT and KO cohorts were not statistically significant except for day 11 (Fig. 2).

For 25 days following single-dose TQ administration, there was no statistical difference between WT and KO cohort mean parasitaemia (Fig. 1). There was no statistically significant difference relative to mean gametocytaemia (Fig. 2) between WT and KO cohorts treated with TQ for the duration of the study. The prolonged duration prior to recrudescence for the TQ-treated animals is likely related to the longer half-life in plasma (WT t_1/2_ = 53.8 ± 3.5 h; CYP2D KO t_1/2_ = 72.4 ± 15.5 h) and delayed clearance relative to PQ [27].

### Pharmacokinetic-pharmacodynamic (PK/PD) analysis

**Fig. 3:**
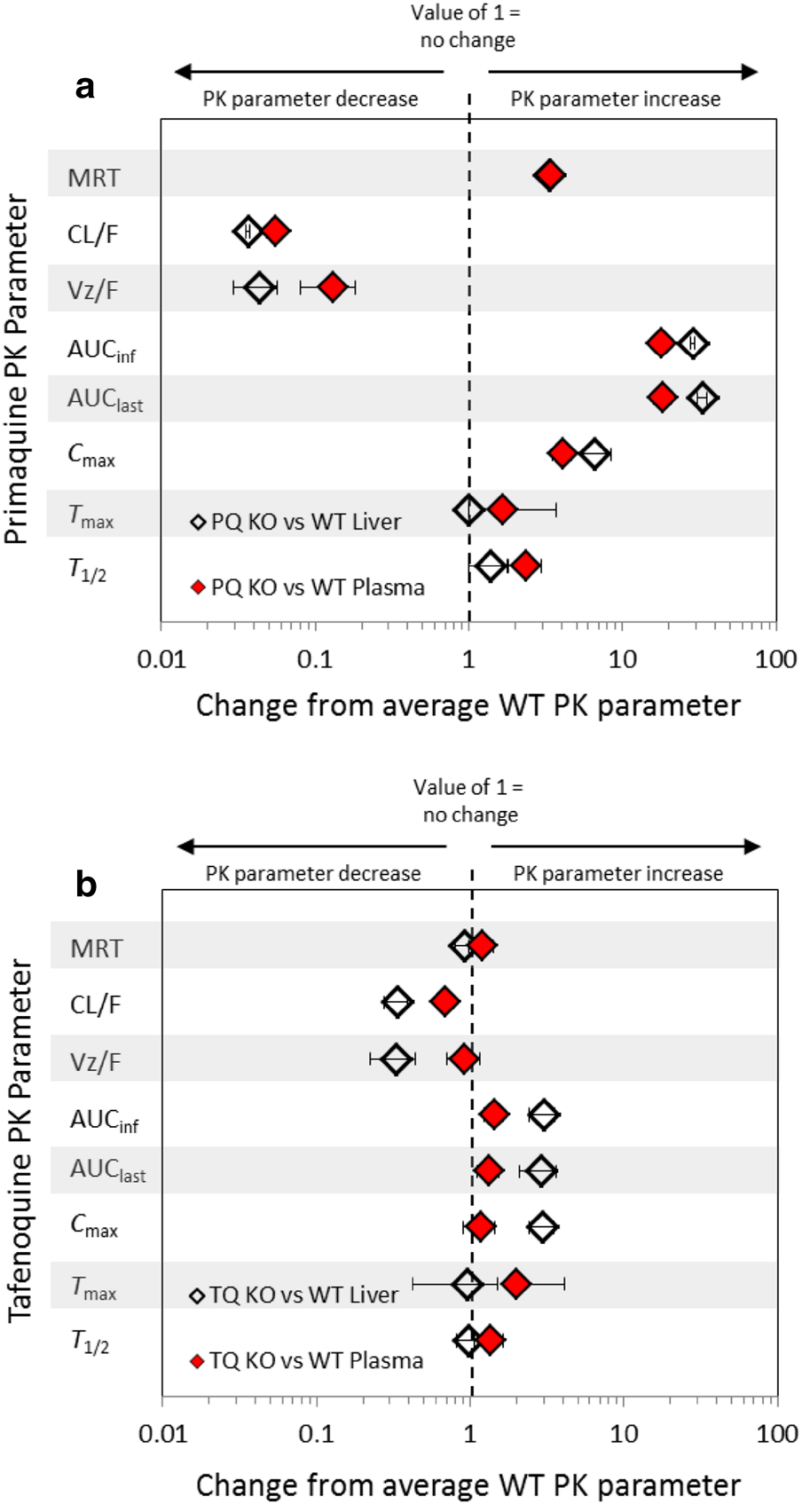
Relative fold changes of primaquine and tafenoquine CYP2D KO pharmacokinetic parameters from reference (WT parameters) in mouse plasma and liver. Indicated are the fold changes after the administration 20 mg/kg primaquine (*PQ*) and 20 mg/kg tafenoquine (*TQ*) in the CYP2D KO strain as compared to WT C57BL/6 mice. The fold change is indicated by the *x-axis* and the pharmacokinetic parameter on the *y-axis*. A value of one represents no relative change in the pharmacokinetic parameter between the KO and WT mice. The *grey bars* are provided for visual reference. The *error* shown is the standard deviation of relative fold changes for KO pharmacokinetic parameters as compared to WT means pharmacokinetic parameter values. PQ and TQ comparisons were conducted using experimental data generated at the WRAIR [26, 27]. *WT* C57BL/6 wild-type mice. *CYP2D KO* C57BL CYP2D6 knock-out mice. *MRT* mean resonance time. *CL/F* apparent total clearance of the drug from plasma after oral administration. *Vz/F* apparent volume of distribution during terminal phase after non-intravenous administration. *AUCinf* area under the plasma concentration-time curve from time zero to infinity. *AUClast* area under the plasma concentration-time curve from time zero to the time of last measurable concentration. Cmax: maximum (peak) plasma drug concentration. *Tmax* time to reachmaximum (peak) plasma concentration following drug administration. *T1/2* elimination half-life

Figure 3 graphically annotates the plasma and liver fold-changes associated with 20 mg/kg PQ (Fig. 3a) and 20 mg/kg TQ (Fig. 3b) CYP2D KO PK parameters versus reference (WT) strain parameters. A value of one represents no relative change in the PK parameter between the KO and WT mice (logarithmic x-axis).

Two items are noteworthy in regards to the fold-changes representing the PK parameters of the parent compound: (1) In contrast to PQ, the mean PK parameters for TQ generally reside toward the central line, which indicates limited relative changes between CYP2D KO and WT mice treated with TQ and (2) both PQ and TQ single-dose administration eradicated erythrocytic parasites despite reduced clearance and increased exposure (AUC) of the parent compound in CYP2D KO mice compared to WT mice. It is important to note that PQ parent exposure is greatly increased in CYP2D KO vs WT mice, whereas TQ exposure is more modestly increased in CYP2D KO vs WT mice.

## Discussion

Three key observations should be highlighted: (1) PQ eradicates hepatic parasites at lower doses than are required to eradicate erythrocytic parasites, while TQ exhibits an analogous dose–response relationship when comparing hepatic and erythrocytic activity, (2) there was no significant difference between WT and CYP2D KO mice regarding the preliminary eradication of erythrocytic parasites (asexual and sexual) following single-dose administration of both TQ (25 mg/kg) and PQ (40 mg/kg), and (3) the variability between WT and CYP2D KO murine PK parameters did not significantly affect the pharmacodynamic endpoints of parasitaemia and gametocytaemia.

PQ is used as a causal prophylactic agent not a blood schizonticidal agent due to relatively greater activity against liver schizonts than blood schizonts [28–31] and in this *P. berghei* murine model, 20 mg/kg PQ causally protected all mice but led to recrudescence when used as a treatment for erythrocytic infection. It may be suggested that this comparability to clinical experience enhances the value of the *P. berghei* murine model and that it may be useful in screening new 8-aminoquinolines and novel drug classes in causal prophylactic and erythrocyte treatment studies.

The somewhat lower prophylactic efficacy of PQ compared to TQ after multiple administrations of both drugs has also been observed clinically. In a review, the efficacy of PQ (approximately 0.5 mg/kg/day from the day prior to parasite exposure until 1 week after parasite exposure) for *P. falciparum* ranged from 85 to 95% in five studies. [1] In contrast, the efficacy of TQ (200 mg/day for 3 days as loading dose followed by 200 mg weekly) was 100% in non-immune Australian troops in comparison to mefloquine efficacy of 100% and placebo failure rate estimated at 8% [32]. TQ efficacy was 98% in semi-immune Africans where the efficacy of mefloquine was also 98% and the placebo failure rate was 32% [33]. This PQ versus TQ comparison supports the PQ hepatic versus erythrocytic infection comparison of the prior paragraph and again suggests that the *P berghei* mouse model may be reasonably comparable to the clinical situation.

If the *P. berghei* model has clinical relevance, our work suggests that CYP2D6 polymorphisms will not be important for TQ erythrocytic clinical activity. The lack of effect due to CYP2D-cluster deletion on TQ erythrocytic activity is in contrast to the previous demonstration of CYP2D-mediated activity relative to liver schizonts in mice [11, 13]. Since TQ prophylactic activity is thought to require some efficacy against blood-stage asexual parasites, the present work suggests that prospective human prophylactic candidates do not need to be screened for 2D6 polymorphisms.

Given that CYP2D-mediated metabolism is required for causal prophylaxis and radical cure (in the case of primaquine) but not for the eradication of the erythrocytic parasites, it is reasonable to suppose that 8-aminoquinolines exhibit different modes of action against different stages of the parasite. Clearing parasites from hepatocytes may involve metabolic activation to oxidative intermediates [11–13], which are produced at lower concentrations in CYP2D KO mice relative to WT mice [26, 27]. In the case of erythrocytic stages, inhibition of haematin agglutination has been proposed [34], although this leads to further questions regarding the mechanistic rational related to the pluripotentiality of 8-aminoquinolines and warrants further exploration.

## Conclusion

Overall, this C57BL murine model mimics the clinical experience for PQ and TQ and may be useful in screening new 8-aminoquinolines and potentially novel drug classes to directly compare causal prophylactic and treatment dosing regimens. The model allowed for the direct comparison of causal prophylaxis and treatment modalities and indicates that PQ inhibits hepatic infections at lower doses than were required to treat erythrocytic infections. In contrast, TQ exhibits analogous dose–response relationships relative to hepatic and erythrocytic infections in wild-type animals. In addition, TQ clears erythrocytic infections at lower doses than PQ. The data comparing wild-type and genetically-modified mice suggest that single-dose administration of TQ and PQ are not impacted by CYP2D mediated metabolism relative to the initial clearing of erythrocytic *P. berghei* infections to the same extent as hepatic infections.

## Additional file

**Additional file 1.** **Figure S1.** Primaquine causal prophylactic (hepatic) IVIS activity (refer to manuscript Table 2). **Figure S2** (Part 1/2 and Part 2/2): Tafenoquine causal prophylactic (hepatic) IVIS activity (refer to manuscript Table 3). **Figure S3**. Comparison of IVIS vehicle control wild-type (WT) and CYP2D knock-out (KO) C57BL mice. **Figure S4**. Individual IVIS bioluminescence signal values measured in C57BL/6 WT and CYP2D KO mice. **Figure S5**. Forrest Plot of percent parasitaemia following 40mpk PQ or 25mpk TQ administered day 4 post IV sporozoite challenge (day 0). **Figure S6**. Forrest Plot of percent gametocytaemia (mature gametocytes) following 40mpk PQ or 25mpk TQ administered day 4 post IV sporozoite challenge. **Figure S7**. Correlation between flow cytometry and light microscopy (based upon percent parasitaemia). Solid line represents linear regression. **Figure S8**. Genetic verification of a subset of C57BL wild-type and CYP2D knock-out mice.
