# Supplementary material for: Cytochrome P450 2D-mediated metabolism is not necessary for tafenoquine and primaquine to eradicate the erythrocytic stages of Plasmodium berghei
Source: Malar J. 2016 Dec 7;15:588. doi: 10.1186/s12936-016-1632-8 (PMC5142148; doi:10.1186/s12936-016-1632-8)

Supplemental Information

Outline:

1. C57BL/6 wild-type (WT) murine causal prophylactic and treatment dose-ranging studies
   1. Figure S1: Primaquine causal prophylactic (hepatic) IVIS activity (refer to manuscript Table 2)
   2. Figure S2 (Part 1/2 and Part 2/2): Tafenoquine causal prophylactic (hepatic) IVIS activity (refer to manuscript Table 3)
2. Comparison of PQ or TQ in wild-type (WT) and CYP2D knock-out (KO) C57BL mice
   1. Figure S3: Comparison of IVIS vehicle control wild-type (WT) and CYP2D knock-out (KO) C57BL mice
   2. Figure S4: Individual IVIS bioluminescence signal values measured in C57BL/6 WT and CYP2D KO mice
   3. Figure S5: Forrest Plot of percent parasitemia following 40mpk PQ or 25mpk TQ administered day 4 post IV sporozoite challenge (day 0)
   4. Figure S6: Forrest Plot of percent gametocytemia (mature gametocytes) following 40mpk PQ or 25mpk TQ administered day 4 post IV sporozoite challenge
   5. Figure S7: Correlation between flow cytometry and light microscopy (based upon percent parasitemia). Solid line represents linear regression
   6. Figure S8: Genetic verification of a subset of C57BL wild-type and CYP2D knock-out mice

_________________________________________________________________________________________________________________________________________________

1. C57BL/6 wild-type (WT) murine causal prophylactic and treatment dose-ranging studies
   1. Primaquine causal prophylactic (hepatic) activity (Table 2)

**Figure S1 (Corresponds to manuscript Table 2). IVIS images at 24 hours, 48 hours, 72 hours. “+” sign corresponds to day of dosing: day -1 (d-1), day 0 (d0), day +1 (d+1). mpk = mg/kg. Primaquine (PQ) = WR002975.**

**
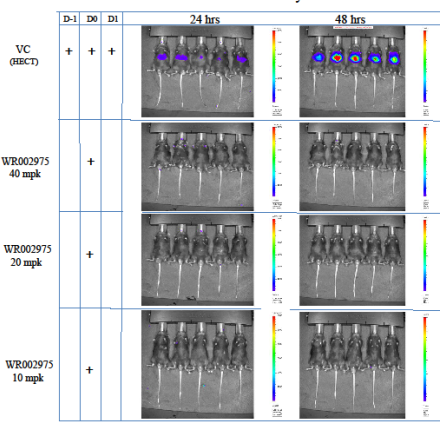

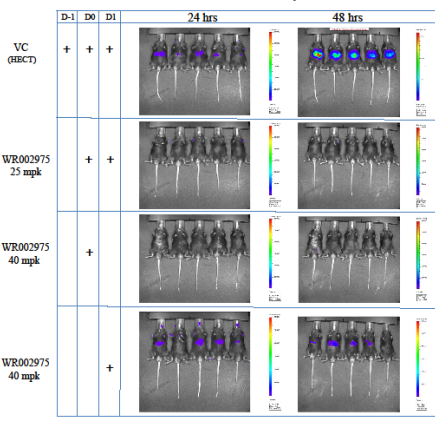
**

- 1. Tafenoquine causal prophylactic (hepatic) activity (Table 3)

**Figure S2 Part 1/2 (Corresponds to manuscript Table 3). IVIS images at 24 hours, 48 hours, 72 hours. “+” sign corresponds to day of dosing: day -1 (d-1), day 0 (d0), day +1 (d+1).. mpk = mg/kg. Tafenoquine (TQ) = WR238605.**


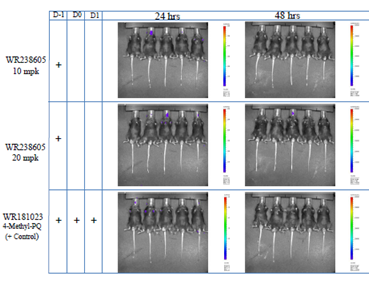

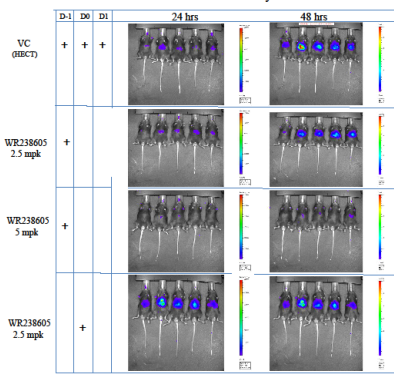


**Figure S2 Part 2/2 (Corresponds to manuscript Table 3). IVIS images at 24 hours, 48 hours, 72 hours. “+” sign corresponds to day of dosing: day -1 (d-1), day 0 (d0), day +1 (d+1). mpk = mg/kg. Tafenoquine (TQ) = WR238605.**

**
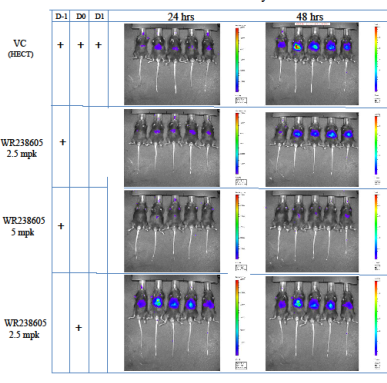

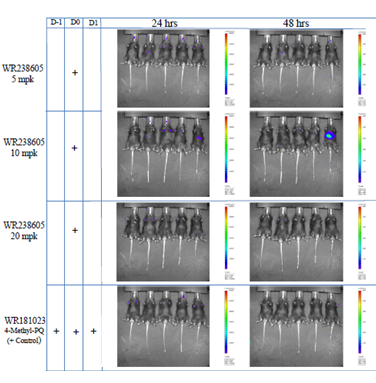
**

1. Comparison of PQ or TQ in wild-type (WT) and CYP2D knock-out (KO) C57BL mice

**Figure S3. IVIS images corresponding to a subset of C57BL wild-type (C57BL WT) and CYP2D knock-out (C57BL CYP 2D KO) vehicle control mice. The images display the disease progression of *P. berghei* following IV sporozoite inoculation (day 0) in WT and KO vehicle control (HECT) mice. Images were captured at 24h and 48h to view the progression of the hepatic infection and 72h to view the subsequent erythrocytic infection:**


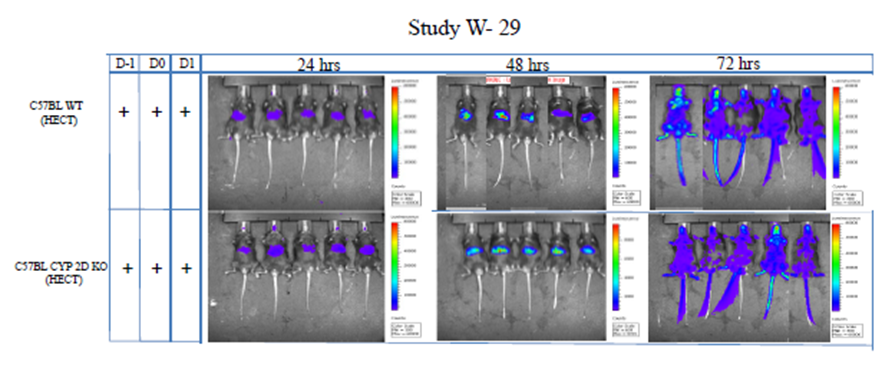


**Figure S4: Individual bioluminescence signal values measured in C57BL/6 WT and CYP 2D KO mice infected IV with 10,000 *P. berghei* sporozoites. Bioluminescent signal, which represents the *P. berghei* parasite load, was measured at (A) 48 hours (liver stage) and (B) 72 hours (blood stage) post infections using the in vivo imaging technology and was expressed in photons/sec. Dots represent individual bioluminescent signal values and bars represent the mean ± SEM from a total of 5 mice for each experimental condition. Unpaired t test with Welch’s corrections (which does not assume equal standard deviations in different experimental conditions) was used to determine whether differences in group means exist.**


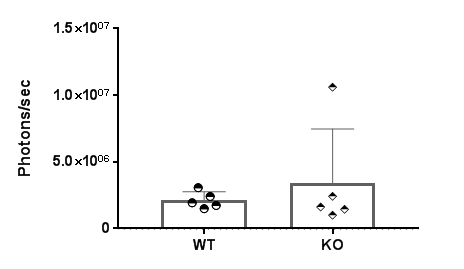


A

B

ns


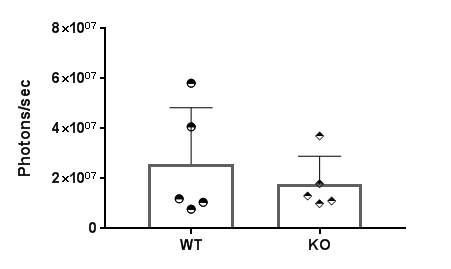


ns

B

**Figure S5. Forrest Plot of percent parasitemia following 40mpk PQ or 25mpk TQ administered day 4 post IV sporozoite challenge (day 0). N = number of animals per cohort. Percent parasitemia quantified using flow cytometry. SEM: standard error of the mean. PQ = primaquine. TQ = tafenoquine. V.C. = vehicle control. WT = C57BL/6 wild-type mice. CYP2D KO = Cytochrome P450 2D knock-out mice.**
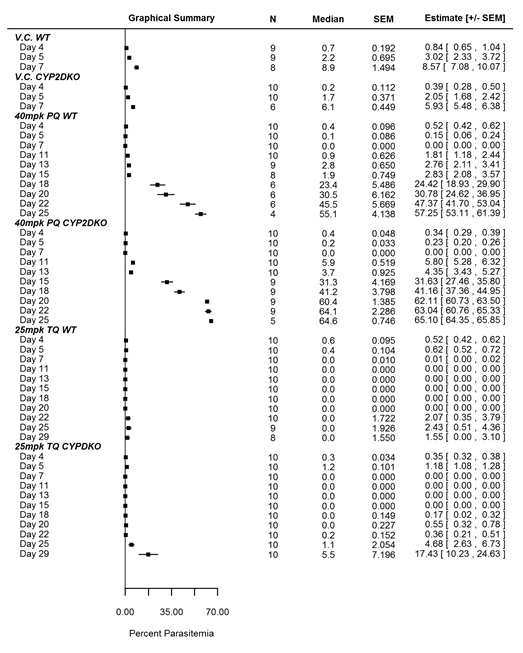


**Figure S6. Forrest Plot of percent gametocytemia (mature gametocytes) following 40mpk PQ or 25mpk TQ administered day 4 post IV sporozoite challenge. N = number of animals per cohort. Percent gametocytemia is derived from light microscopy parasite counts. SEM: standard error of the mean. PQ = primaquine. TQ = tafenoquine. V.C. = vehicle control. WT = C57BL/6 wild-type mice. CYP2D KO = Cytochrome P450 2D knock-out mice.**


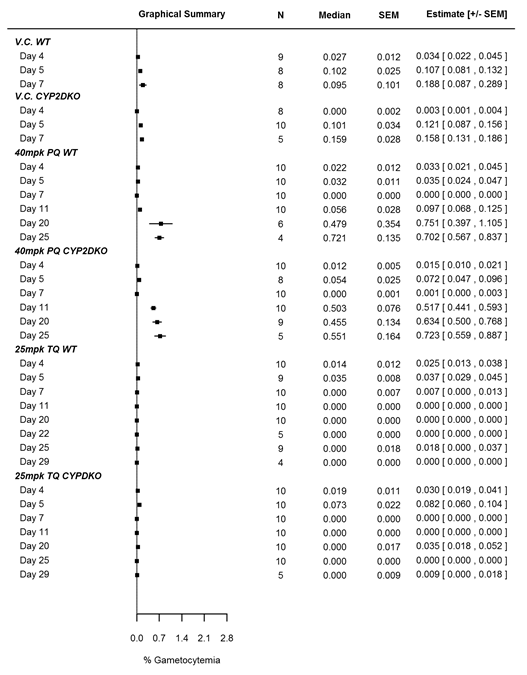


**Figure S7. Correlation between flow cytometry and light microscopy (based upon percent parasitemia). Solid line represents linear regression. Dotted line represents a third-degree polynomial. Dashed line represents LOESS curve is shown with a dashed line.**


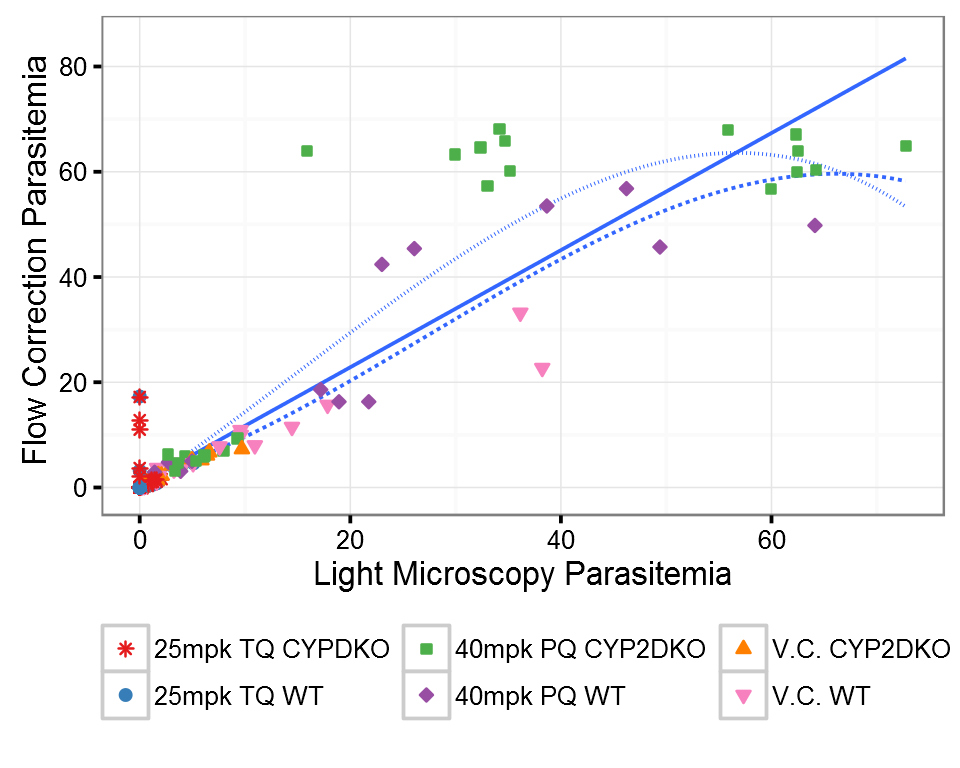


**Figure S8. Genetic verification of a subset of C57BL wild-type and CYP2D knock-out mice:**


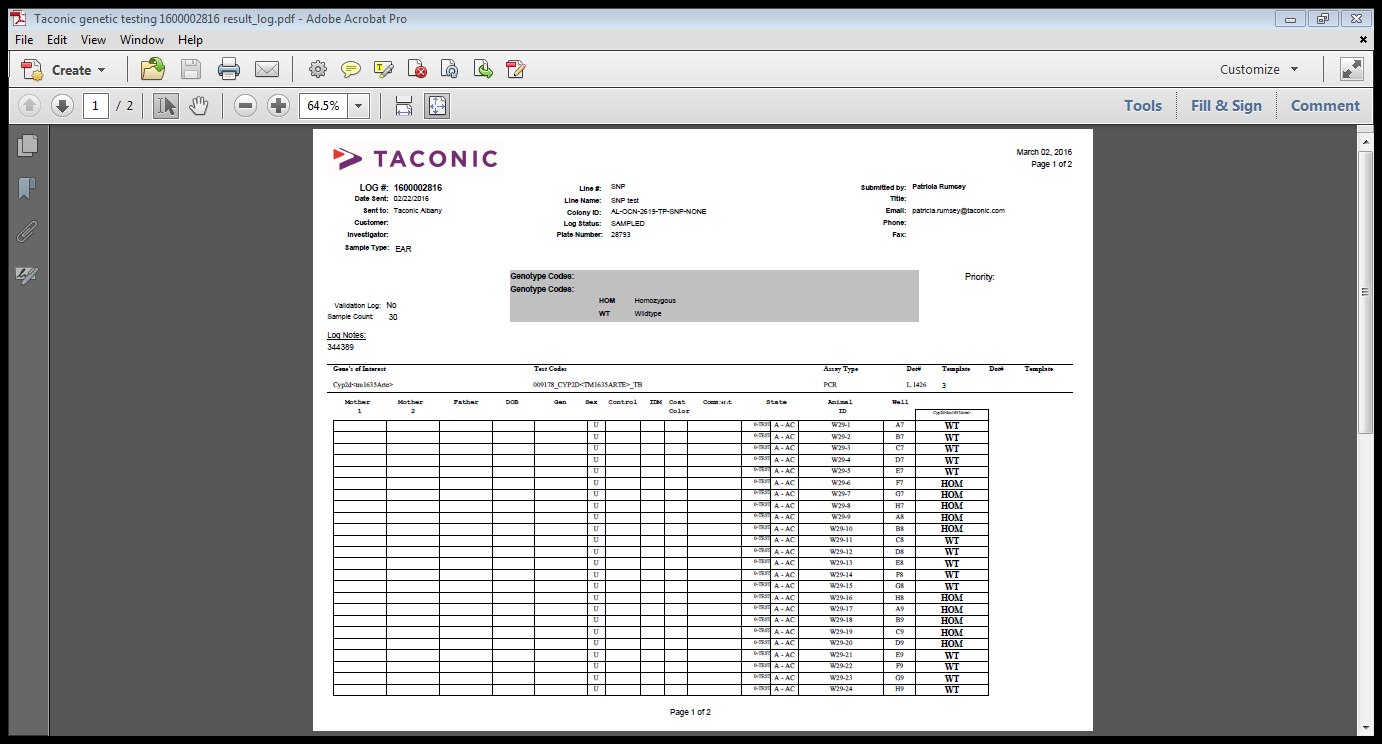


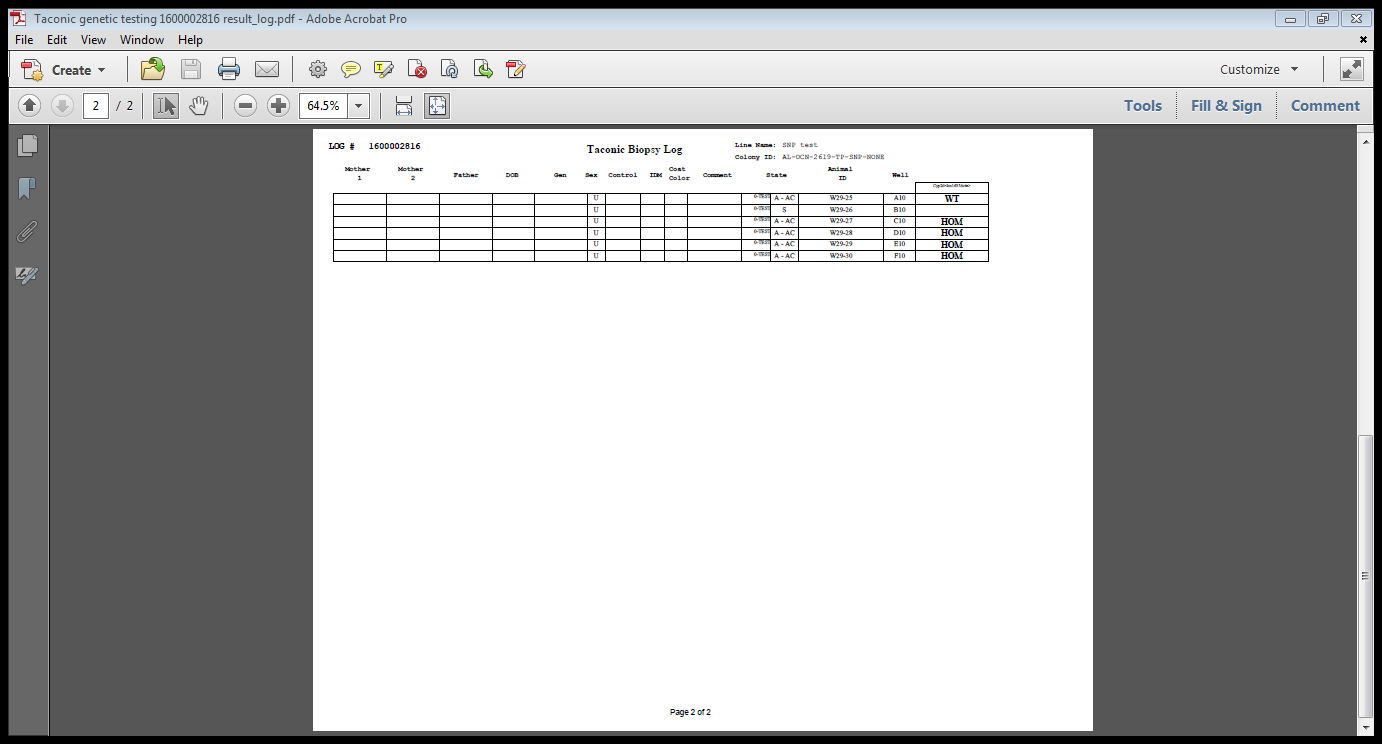

Supplement: Supplementary file 1 — Additional file 1. Figure S1. Primaquine causal prophylactic (hepatic) IVIS activity (refer to manuscript Table 2). Figure S2 (Part 1/2 and Part 2/2): Tafenoquine causal prophylactic (hepatic) IVIS activity (refer to manuscript Table 3). Figure S3. Comparison of IVIS vehicle control wild-type (WT) and CYP2D knock-out (KO) C57BL mice. Figure S4. Individual IVIS bioluminescence signal values measured in C57BL/6 WT and CYP2D KO mice. Figure S5. Forrest Plot of percent parasitaemia following 40mpk PQ or 25mpk TQ administered day 4 post IV sporozoite challenge (day 0). Figure S6. Forrest Plot of percent gametocytaemia (mature gametocytes) following 40mpk PQ or 25mpk TQ administered day 4 post IV sporozoite challenge. Figure S7. Correlation between flow cytometry and light microscopy (based upon percent parasitaemia). Solid line represents linear regression. Figure S8. Genetic verification of a subset of C57BL wild-type and CYP2D knock-out mice. [file 12936_2016_1632_MOESM1_ESM.docx]
